# Culture, prefrontal volume, and memory

**DOI:** 10.1371/journal.pone.0298235

**Published:** 2024-03-29

**Authors:** Nicolette Barber, Ioannis Valoumas, Krystal R. Leger, Yu-Ling Chang, Chih-Mao Huang, Joshua Oon Soo Goh, Angela Gutchess

**Affiliations:** 1 Department of Psychology, Brandeis University, Waltham, MA, United States of America; 2 Graduate Institute of Brain and Mind Sciences, College of Medicine, National Taiwan University, Taipei, Taiwan; 3 Neurobiology and Cognitive Science Center, National Taiwan University, Taipei, Taiwan; 4 Center for Artificial Intelligence and Advanced Robotics, National Taiwan University, Taipei, Taiwan; 5 Volen National Center for Complex Systems, Brandeis University, Waltham, MA, United States of America; 6 Department of Biological Science and Technology, National Yang Ming Chiao Tung University, Hsinchu, Taiwan; 7 Institute of Brain Science, National Yang Ming Chiao Tung University, Taipei, Taiwan; 8 Department of Psychology, National Taiwan University, Taipei, Taiwan; University of Bari Department of Education Psychology and Communications: Universita degli Studi di Bari Aldo Moro Dipartimento di Scienze della Formazione Psicologia Comunicazione, ITALY

## Abstract

Prior cross-cultural studies have demonstrated differences among Eastern and Western cultures in memory and cognition along with variation in neuroanatomy and functional engagement. We further probed cultural neuroanatomical variability in terms of its relationship with memory performance. Specifically, we investigated how memory performance related to gray matter volume in several prefrontal lobe structures, including across cultures. For 58 American and 57 Taiwanese young adults, memory performance was measured with the California Verbal Learning Test (CVLT) using performance on learning trial 1, on which Americans had higher scores than the Taiwanese, and the long delayed free recall task, on which groups performed similarly. MRI data were reconstructed using FreeSurfer. Across both cultures, we observed that larger volumes of the bilateral rostral anterior cingulate were associated with lower scores on both CVLT tasks. In terms of effects of culture, the relationship between learning trial 1 scores and gray matter volumes in the right superior frontal gyrus had a trend for a positive relationship in Taiwanese but not in Americans. In addition to the a priori analysis of select frontal volumes, an exploratory whole-brain analysis compared volumes—without considering CVLT performance—across the two cultural groups in order to assess convergence with prior research. Several cultural differences were found, such that Americans had larger volumes in the bilateral superior frontal and lateral occipital cortex, whereas Taiwanese had larger volumes in the bilateral rostral middle frontal and inferior temporal cortex, and the right precuneus.

## Introduction

Prior research has demonstrated the potential for different types of learning and experiences to alter the brain’s wiring and structure [1–3]. In terms of memory, neuroanatomical differences across individuals could affect how well information is encoded, consolidated, and stored in memory, or, inversely, having a stronger or poorer ability to remember information can sculpt the cortical thickness, volume, and surface area of the brain. In the present study, we focus on the relationship between performance on a verbal learning task and corresponding differences in structural measures of prefrontal volumes. In particular, neuropsychological tasks assessing memory, including the California Verbal Learning Test [4] have been shown to be sensitive to structural volumes [5]. In addition, we focus on individual differences through the lens of culture, comparing participants from the United States and Taiwan to assess the ways in which relationships between structural measures of the brain and performance on tests of memory may differ across cultural groups.

### Cultural influences on cognition and neural structures

Culture represents one set of life experiences that can shape cognition and the brain [6]. In terms of attention and memory, East Asians have a holistic processing orientation that includes focusing broadly, such as attending to the entire field or considering the relationship between an object and its context; in contrast, Westerners have an analytic processing orientation that is associated with narrower object focus and organizing information by rules and categories independent of context [7–9]. These different orientations lead Americans to have more detailed autobiographical memory [10] and higher levels of specific memory for object details than East Asians [11, 12]. Easterners tend to focus on functional relationships between items whereas Westerners focus on hierarchical organization such as taxonomic categories [13, 14]. In addition, one study using the Framed-Line test illustrated this dissociation in processing styles, with Americans more accurate at drawing the line in the absolute task, indicating better memory for exact size of focal objects, whereas East Asians were more accurate for the proportional task, indicating better memory for contextual relationships [15, 16] Some research has linked cultural differences in holistic and analytic processing styles to independent and interdependent self-construal styles [17]. Western cultures promote an independent self-construal, focusing on the self as distinct from others, appreciating one’s differences compared to others, and valuing asserting oneself [7, 18]. East Asian cultures promote an interdependent self-construal, conceptualizing of the self in relation to others, focusing on fitting in with others, and stressing the importance of harmonious relationships [7, 18].

Evidence for cultural differences also emerges in comparisons of cognitively impaired populations. Chinese and Americans were compared on a neuropsychological assessment called the Blessed-Roth Information-Memory-Concentration Test [19]. Chinese participants outperformed the Americans in answering questions regarding orientation to time and place, suggesting a more holistic orientation emphasizing context. However, the Americans performed better on items that required more analytic detail-focus such as recall of specific historical dates [20]. These results indicate the pervasiveness of cultural differences, even having implications for neuropsychological assessment of patients [21].

In terms of literature on cross-cultural differences in the structure of the brain, there are merely a handful of studies that have compared Easterners and Westerners. One study collected MRI measures of cortical thickness and density from younger and older Singaporeans of Asian descent and Americans [22]. The participants were well matched for neuropsychological performance in several domains. Results indicated that several frontal regions and the right parietal lobule were larger in younger Americans than Singaporeans, although the left temporal gyrus was thicker in Singaporeans than Americans [22]. Another study converged with Chee et al [22]’s results in finding that structures within the frontal and parietal lobe were smaller in Chinese compared to Caucasians [23, 24]. However, this study also indicated that Chinese participants had greater cortical volume, thickness and surface area in several specific temporal lobe structures and the paracingulate/cingulate gyrus compared to Caucasians [23]. In addition, Huang et al [25] found consistent results in that frontal-parietal areas and the cerebellum are larger in Westerners, whereas temporal-occipital regions are larger in Easterners [25]. It has been suggested that the thickness in the prefrontal/frontal areas found in the American group could be due to the increased emphasis that this culture puts on independent thinking and analytical processing, whereas East Asians process information more holistically [16, 22].

Although these studies document differences in structural regions across Americans and East Asians, the literature is sparser in terms of linking these differences in structure to performance on behavioral task, beyond consideration of language (e.g., [26, 27]). Some studies investigate specific regions associated with specific processes. For example, larger gray matter volumes in the parahippocampal place areas for East Asians than European Americans are thought to reflect scene processing [28]. Larger volume in the temporo-parietal junction for East Asians compared to European Americans is thought to reflect cultural differences associated with perspective taking and mentalizing [29]. Considering interdependent vs independent self-construal styles, higher independence scores have been associated with larger gray matter volume in the ventral medial prefrontal cortex (vmPFC) [30, 31], right dorsolateral prefrontal cortex (dlPFC), right rostral lateral prefrontal cortex (rlPFC) [30], and the orbitofrontal prefrontal cortex (OFC) [31, 32]. Higher interdependence scores were linked to larger grey matter volume in the right TPJ [29], and reduced OFC volume [31, 32].

### Relationship between prefrontal regions and neuropsychological test performance

Although research linking cultural differences in brain structure to performance on standardized neuropsychological tasks is rare, there is more literature that considers these relationships without culture. Episodic memory has been associated with several neural areas, and the present study will focus on regions of prefrontal cortex. The California Verbal Learning Test (CVLT) is a commonly used neuropsychological measure assessing long-term memory [4]. The task invokes many processes, including organization (semantic and subjective), context memory, memory search (recall and recognition) and response bias (yes/no recognition) [33]. Frontal regions have been implicated in task performance; frontal lobe damage is associated with impaired list learning and poor free recall performance on the CVLT [33, 34]. Interactions between the medial temporal lobes (MTL) and the frontal lobes are crucial for normal memory function [33], with prefrontal regions supporting strategic processes and the supervision and selection of appropriate strategies in memory (e.g., categorization in the CVLT; [34]. Patients with injuries in the frontal lobe are impaired in this organizational skill, but when given instructions to apply the strategy, they performed normally on recall tests [34–37].

Aside from studies with patients, volumetric differences in PFC regions in healthy control participants may reflect differences in memory strategies. Thickness in the dorsolateral prefrontal cortex (dlPFC) has been associated with memory performance on the delayed recall portion of the CVLT [38]. This region is involved in the formation of long-term memory (LTM) through strengthening associations among items in working memory [39]. However, another study implicated frontal regions in organization, finding that reductions in volume of the left superior and inferior frontal lobe and right dlPFC were associated with increased semantic clustering on the CVLT [40], whereas increased activity in the left PFC was associated with recognition of familiar words on the CVLT [41]. Cortical thickness of the anterior midcingulate cortex extending into the paracingulate cortex and rostral medial prefrontal cortex have also been associated with higher scores on the delayed recall portion of the CVLT [38]. Moreover, damage to the medial prefrontal cortex (mPFC) has been associated with impairment in performance on the CVLT [42, 43]. The integrity of the anterior midcingulate, a subdivision of the anterior cingulate cortex [44], has also been positively correlated with cognitive control, which in turn enhances memory performance [45, 46]. This region contributes to allocating of control resources appropriately to a given task [47]. The present study focuses on the volume of these prefrontal regions, based on the past findings of associations of these regions with CVLT performance.

### Linking the CVLT to cultural brain differences

Culture may be one source of individual differences that impacts how volumes of brain regions relates to performance on the CVLT. As Easterners and Westerners differ in their use of cognitive strategies and their recruitment of brain networks, this could impact the volume of brain structures (e.g., [22, 23, 25]). Notably, several of the brain areas showing cultural differences in structure are also associated with performance on the CVLT as well as more broadly in memory encoding and retrieval. These broadly include the dlPFC, mPFC, and cingulate cortex. The vmPFC was found to be larger in Westerners [30, 31]. Damage to this area and the basal forebrain have been associated with impairment on the CVLT due to deficits in drawing direct and indirect relationships between elements [48]. Another structure found to be larger in Westerners is the rostral medial PFC (rmPFC) [22], a region that was also linked to increased performance on the CVLT [38]. The anterior midcingulate cortex is associated with higher scores on the CVLT [38], an area near the cingulate regions that was larger in East Asians than Westerners [23]. This region also has functional connections to the paracingulate [46], another brain region often observed to be larger in East Asians [23]. Finally, in general it was found that the dlPFC tends to be larger in Westerners [30]. Volume of the dlPFC is highly variable in terms of individual memory strategies and performance on the CVLT [38, 40]. Overall, differences within these regions may underlie cultural differences in CVLT performance, reflecting differences in orientation and memory strategies across cultures [49]. This is consistent with the fact that individual differences in brain activity and memory performance reflect differences in self-initiated encoding strategies [50].

#### Predictions

In this study, we first investigate the relationship between CVLT scores and gray matter volumes, without regard to culture. We chose to focus on volume because the measure takes into account both cortical thickness and surface area [51], and the measure is generally more reliable than cortical thickness alone [52]. For hypothesis 1 (H1), we predict that higher scores on the CVLT will be related to larger gray matter volumes in the superior frontal and rostral middle gyrus (dlPFC), the lateral orbitofrontal and medial orbitofrontal gyri (vmPFC), the rostral anterior cingulate (rmPFC) and the caudal anterior cingulate (anterior midcingulate cortex), as these areas have been implicated in memory performance on the CVLT and show a wide degree of morphological variation in terms of memory strategies. Second, we investigate whether culture modifies the relationship between CVLT scores and these gray matter volumes. For hypothesis 2 (H2), we predict that there will be cultural differences within the above stated brain regions associated with scores on the CVLT. These predictions are motivated based on previous findings of cultural differences in gray matter volumes likely to be implicated in CVLT performance. To converge with prior studies that compared the volume of regions across cultural groups [22–25] without considering the relationship with CVLT scores, we will also conduct exploratory analyses comparing volumes of cortical regions across cultures.

## Methods

### Participants

A total of 115 Taiwanese and US young adults, ages 18–30, completed the study between August 2019 and August 2022 and were included in the study. All participants were right-handed and had no previous history of neurological or psychological disorders. Fifty-seven participants (28 females; 29 males) were Taiwanese young adults, with an average age of 23.26 (*SD =* 2.40). They were recruited from the National Taiwan University (NTU) and Taipei City area in Taiwan. Fifty-eight participants (30 females, 28 males) were US young adults, with an average age of 21.31 *(SD =* 3.23). They were recruited from Brandeis University and the surrounding Boston area. All participants were native to their respective country and had not lived outside of their country for more than two years. Each participant provided written informed consent before the start of this study. Protocols (#19034r) were approved by the Brandeis University Institutional Review Board and NTU Hospital Research Ethics Committee. Participants were compensated for their time. Although data had alphanumeric codes, primary experimenters had access to information that could identify individual participants during and after data collection.

### Neuropsychological assessment

A battery of neuropsychological tests was administered to all participants in their native language. The specific neuropsychological measure that was the focus of this analysis was the California Verbal Learning Task II (CVLT-II) [53]. This is a commonly used neuropsychological test to measure episodic verbal learning and memory [54]. During learning, the experimenter reads a list of 16 words from 4 semantic categories (List A). The words were repeated over five learning trials; after each iteration the participant was asked to recall as many words as possible. An interference list trial (List B) was read immediately after the fifth trial, and participants were asked to recall as many words as they could remember from List B only. Following this, the participants were asked to recall the items on List A in short and delayed (approx. 20 minutes) and cued recall trials. In the cued recall trials, specific categories were given (animals, furniture, travel, and vegetables) and participants were to recall the words from List A that fit into those categories. Lastly, participants completed a delayed recognition test. For the present study, the outcome variables of interest for this study are the raw scores on a) learning trial 1 and b) long delayed free recall. Performance on these measures have been related to the selected brain structures in previous literature [38, 41]. Scores on trial 1 may also relate to scores on the long delayed free recall trials, indicating encoding differences [55]. In addition, cultural groups differed the most on learning trial 1 (see Results; S1 Table includes scores and exploratory comparisons across the cultural groups on the remainder of the CVLT measures).

### Brain imaging acquisition

MRI data was collected using identical 3T Siemens MAGNETOM Prisma systems with 64 channel head coils located at the Imaging Center for Integrated Body, Mind and Culture Research, National Taiwan University, Taipei, Taiwan, and the Center for Brain Science, Neuroimaging facility, Harvard University, Cambridge, MA, USA. Calibration analyses were conducted prior to data collection, testing the same individuals on both scanners in order to establish the comparability of functional data across the scanners. Results showed that global signal did not differ across scanners and activation differences only occurred in visual cortex, consistent with differences in the luminance of the screen [56]. A standardized high resolution T1-weight magnetization-prepared rapid gradient echo image (multi-echo MPRAGE: [57]) was obtained for gray-white matter with 176 sagittal slices (voxel size 1.0 × 1.0 × 1.0 mm), FOV = 256 × 256 mm, TR = 2530.0 ms, short TE = 1.69 ms, long TE = 7.27 ms, and FA = 7°.

### Analysis of structural MRI data

All MRI data was analyzed using FreeSurfer 6.0.0 (http://surfer.nmr.mgh.harvard.edu/); this text is based on standard methods language provided by FreeSurfer. Imaging processing included motion correction, averaging [58] of multiple volumetric T1 weighted images, skull-stripping [59], Talairach transformation, segmentation of the subcortical white/gray matter volumetric structures [60, 61], intensity normalization [62], tessellation of the gray/white matter boundaries, topology correction [63, 64], and surface deformation to optimally place the gray/white and gray/cerebrospinal fluid borders [65–67]. Once reconstruction was complete, the cerebral cortex was parcellated in respect to individual gyral and sulcal patterns [61, 68]. This method uses both intensity and continuity information from the entire three-dimensional MR volume in segmentation and deformation procedures to produce representations of cortical thickness, calculated as the closest distance from the gray/white boundary to the gray/CSF boundary at each vertex on the tessellated surface [67]. Procedures for the measurement of cortical thickness have been validated against histological analysis [69] and manual measurements [70, 71]. Freesurfer morphometric procedures have been demonstrated to show good test-retest reliability across scanner manufacturers and across field strengths [72, 73]. Automatic parcellations were visually inspected and manually corrected for each of the participants.

### Analysis of Regions-of-Interest (ROI)

The Desikan-Killiany-Tourville (DKT) atlas [74] was used for volumetric measurements. We chose regions in accordance with the DKT atlas protocol that best represented the regions implicated in task performance in prior studies. In total, we had twelve ROIs (six in each hemisphere). Of the region options available in the DKT atlas we chose the rostral middle and superior frontal gyri, corresponding to the dlPFC. In addition, we selected the lateral orbitofrontal and medial orbitofrontal gyri, corresponding to the vmPFC. These ROI options available through FreeSurfer best represented the regions described in the literature; each ROI was analyzed separately rather than combined into a larger region (e.g., left lateral and medial orbitofrontal gyri were two separate ROIs rather than combined into one left vmPFC ROI). Further, the rostral anterior cingulate (rACC) was selected as the region that best encompassed the location of the rmPFC as discussed in Sun et al [38]. Finally, the caudal anterior cingulate (cACC) was chosen to best represent the anterior midcingulate.

### Analytic plan

Analyses of the *a priori* ROIs were preregistered: https://aspredicted.org/SVP_CK1. Since the pre-registration, data from an additional 7 American participants were collected and included in the analyses. Initially, we only conducted the analyses that were pre-registered to test hypotheses (i.e., all analyses are reported in the manuscript). Additional exploratory analyses are included in order to more fully characterize the dataset (e.g., exploratory tests of the relationship between CVLT scores and gray matter volume across the whole brain; comparisons of cortical volumes without regard to CVLT performance; all CVLT scores). These are labeled as “exploratory”, and not incorporated into the discussion section. The data were collected as part of a larger project studying cognition and neural activity across cultures [75–77]. The sample size was based on estimates needed for the primary fMRI study [76]. All young adult participant data available were included in the present analyses of structural MRI data. Data are available at: https://osf.io/zfd9e/

Outliers were defined as values that were outside a range of 3 standard deviations. Eight participants were identified as outliers based on their scores and volumes (i.e., for values on CVLT learning trial 1, CVLT long delayed free recall (LDFR), left and right superior frontal gyrus, left and right cACC). Scores and volumes deemed outliers were only removed from analyses that included the outlier values, but the participants were included in all other analyses. Outliers were not identified or removed for exploratory analyses.

Memory scores on the CVLT learning trial 1 and long delayed free recall (LDFR) were analyzed using an independent two tailed t-test to determine whether there were cultural differences in memory performance. Exploratory analyses comparing volumes of brain regions across cultures were conducting using two tailed independent samples t-tests.

The relationships between prefrontal gray matter regions and memory scores were tested using linear regression analyses. For Hypothesis 1, we were interested in the relationship between the volume of ROIs and memory scores. For Hypothesis 2, we then addressed if culture affected the relationship between the volume of ROIs and memory scores. Separate linear regressions were conducted with learning trial 1 and LDFR as dependent variables and the ROI volumes as independent variables. Although we did not pre-register it, we also ran analyses with sex as a covariate. All the significant effects reported here persist when sex is included as a covariate in the analyses. It was also necessary to adjust for head size by accounting for intracranial volume (ICV) in the analyses. We had initially intended to include ICV as a covariate in the two models; for H1: CVLT score = gray matter volume + ICV and H2: CVLT score = culture * gray matter volume + ICV respectively. However, the analyses revealed that ICV effects differed across analyses depending on brain region. For this reason, deviating from the original pre-registration, we opted to first derive ICV adjusted ROI volumes to account for the differential influence of head size specific to each ROI [78, 79], each ROI was adjusted using the same global average ICV (i.e., across all participants), as in a previous cross-cultural study [22]. Specifically, we adjusted ROI volumes using the following equation:

$$Volume_{Adj}=Volume_{Raw}-b(ICV–Mean\;ICV)$$

Where *b* is the slope of the linear regression between Volume_Raw_ and ICV. The adjusted volumes for each region were then used in analyses. Two regressions were conducted for each of the two dependent variables and an interaction term for ROIs x Culture for each corresponding brain region was computed.

#### Exploratory comparisons of volumes across cultures

We supplemented our analyses focused on the gray matter volumes we predicted would be associated with CVLT performance by conducting exploratory analyses comparing volumes of other cortical regions across cultures both with the CVLT to assess memory performance and without. The analyses that do not consider performance on the CVLT allow for comparisons of our samples with findings from prior studies focused only on comparisons of volume across cultures [22–25], as well allowing for tests of the robustness of cultural differences. To do this, we selected a whole brain vertex-based analysis approach. Analyses were conducted using Freesurfer’s mri_glmfit to test for the relationship between culture and gray matter volume. Note that for this analysis, Taiwanese participants were coded as 1, Americans were coded as -1. Surfaces were resampled into a common space (fsaverage) and smoothed with a 10mm full width half maximum kernel (FWHM). Whole brain analyses were corrected for multiple comparisons using Monte Carlo simulations with cluster forming threshold of p < .0001, and cluster wise p < .05. ICV centered was used as a nuisance variable in this analysis.

## Results

### CVLT performance

We first assessed cultural differences in memory performance on CVLT learning trial 1 and long delayed free recall (LDFR) scores. The American participants’ performance was higher than Taiwanese participants’ on learning trial 1 (US: *M* = 8.35, *SD* = 2.10; Taiwanese: *M* = 7.28; *SD* = 2.18), and this difference reached significance (*t*(112) = 2.66, *p* < .01, Cohen’s *d* = 2.14). However, there was no significant difference on the LDFR (*t*(111) = .38, *p* = .70), with both groups performing similarly (US: *M* = 14.30, *SD* = 1.80; Taiwanese: *M* = 14.16, *SD* = 1.99). Note that high performance on the measure may limit the sensitivity of this measure due to restricted range.

### Comparison of brain volumes across cultures

In comparing the gray matter volume for each of the 12 pre-registered prefrontal regions, using the volumes adjusted for ICV, there was a significant difference between cultures within the bilateral superior frontal gyrus, bilateral rostral middle gyrus and the right rostral anterior cingulate. Results are shown in Table 1. Taiwanese young adults had smaller left and right superior frontal volumes compared to US young adults. In contrast, Taiwanese young adults had larger left and right rostral middle frontal and larger right rACC volumes than Americans.

**Table 1:** Independent samples t-test of cultural differences in the gray matter volume of brain regions.

Volume (mm^3^)		Culture	*N*	*M*	*SD*	*t*	*p*
	L	US	57	9494.08	623.09	0.08	0.93
Lateral Orbitofrontal		TW	57	9483.14	770.11		
	R	US	57	9031.57	767.44	-1.32	0.18
		TW	57	9220.16	753.97		
	L	US	57	12203.18	1415.61	-2.81	< .01**
Rostral Middle Frontal		TW	57	12917.88	1292.44		
	R	US	57	11944.24	1651.61	-2.70	< .01**
		TW	57	12689.61	1266.2		
	L	US	56	26325.35	1805.29	2.49	0.01*
Superior Frontal		TW	57	25469.35	1833.59		
	R	US	56	28978.34	1727.23	3.51	< .01**
		TW	57	27809.11	1807.92		
	L	US	57	4776.29	396.25	-1.83	0.06
Medial Orbitofrontal		TW	57	4916.75	421.25		
	R	US	57	4557.85	449.35	-0.02	0.98
		TW	57	4559.9	413.08		
	L	US	57	3697	404.47	-1.88	0.06
Rostral Anterior		TW	57	3845.82	439.4		
Cingulate Cortex	R	US	57	2522.7	396.02	-2.56	0.01*
		TW	57	2720.53	427.93		
	L	US	56	3220.12	371.75	-1.22	0.22
Caudal Anterior		TW	57	3314.79	446.04		
Cingulate Cortex	R	US	57	2198.17	447.82	-1.30	0.19
		TW	55	2311.98	478.49		

Note: Sample sizes vary due to the exclusion of outliers; US = Americans; TW = Taiwanese

*Indicates significance at p < .05

### Relationship between prefrontal volumes and CVLT performance

**Fig 1:**
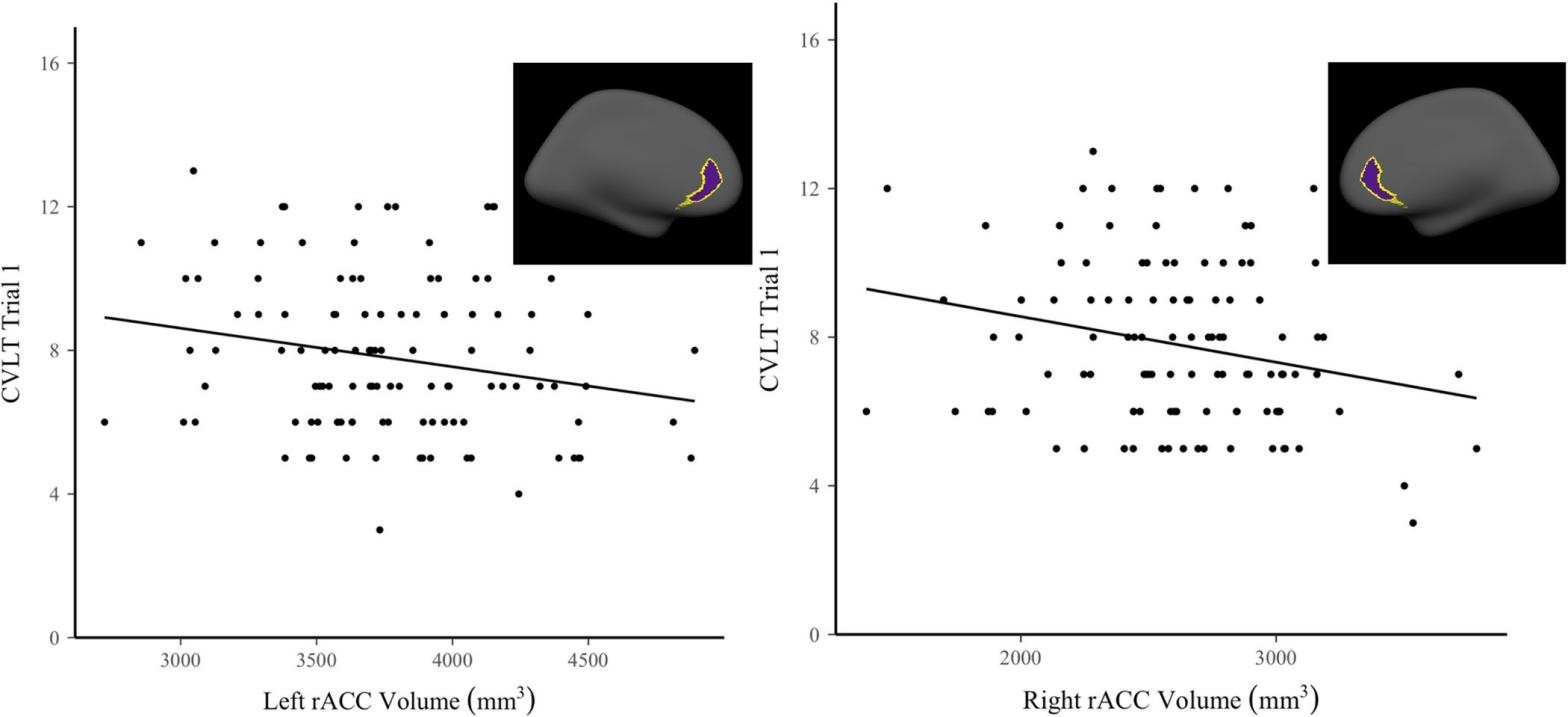
Relationship between left and right rACC volume and performance on CVLT trial 1.

We next analyzed the association between prefrontal volumes and memory performance, corresponding to our first hypothesis. Linear regressions using the adjusted volumes were run with CVLT learning trial 1 scores and LDFR scores as the outcomes. The results revealed a significant relationship between the bilateral rostral anterior cortex and learning trial 1 scores. Larger volumes in the left and right rACC regions were associated with lower scores on trial 1 of the CVLT, as displayed in Fig 1. No other significant effects were found for trial 1 or LDFR scores; see Table 2 for all results. The overall R^2^ for both models were .08 and .05 respectively.

**Table 2:** Regression analysis: Relationship between prefrontal volumes and memory Outcomes (H1).

Volume (mm^3^)			Trial 1			LDFR	
		β	*t*	*p*	β	*t*	*p*
Lateral Orbitofrontal	Left	0.08	0.85	0.39	0.15	1.64	0.10
	Right	0.13	1.39	0.16	0.11	1.19	0.23
Rostral Middle Frontal	Left	-0.08	-0.9	0.36	-0.02	-0.29	0.76
	Right	-0.11	-1.18	0.24	-0.05	-0.57	0.57
Superior Frontal	Left	0.15	1.58	0.11	0.15	1.65	0.10
	Right	0.1	1.06	0.29	0.12	1.25	0.21
Medial Orbitofrontal	Left	-0.07	-0.8	0.42	0.07	0.79	0.42
	Right	-0.11	-1.22	0.22	0.05	0.57	0.56
Rostral Anterior	**Left**	**-0.21**	**-2.26**	**0.02** *	0.08	0.88	0.37
Cingulate Cortex	**Right**	**-0.23**	**-2.58**	**0.01** *	-0.14	-1.48	0.14
Caudal Anterior	Left	-0.14	-1.49	0.14	-0.07	-0.73	0.46
Cingulate Cortex	Right	0.02	0.21	0.83	0.07	0.8	0.42

* Indicates significance at p < .05

### Cultural differences in the relationship between brain volume and CVLT performance

**Fig 2:**
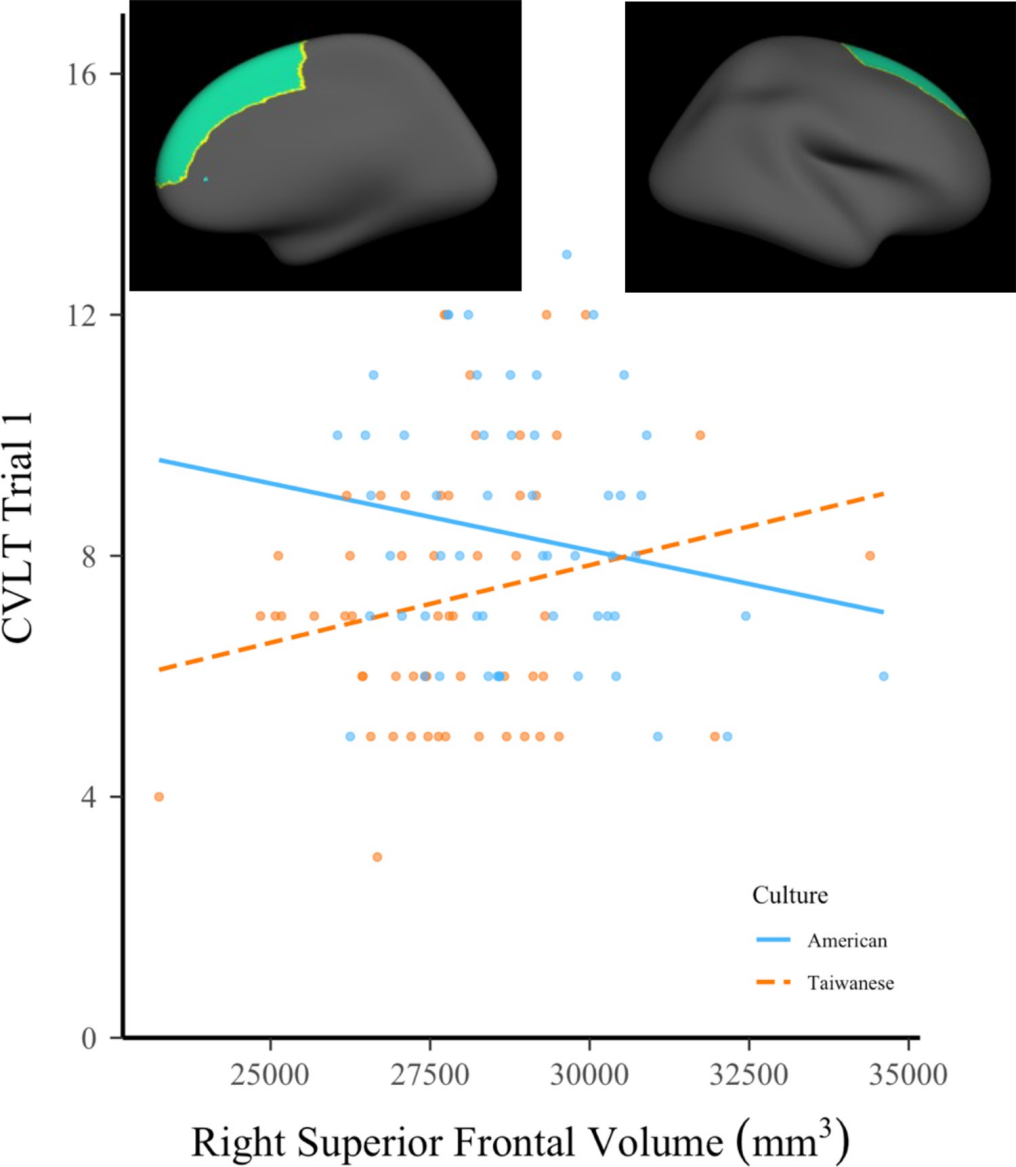
Interaction between culture and right superior frontal volume and performance on CVLT trial 1.

We next examined the interaction between culture and the gray matter volume of regions on CVLT trial 1 and LDFR scores. For trial 1, there was a significant main effect of culture on memory performance, and a significant interaction between culture and right superior frontal gyrus; see Table 3. The interaction is shown in Fig 2. To further understand this interaction, we calculated correlations between CVLT trial 1 scores and volume of the right superior frontal gyrus for each cultural group (Taiwanese: *r*(57) = .24, *p* = .08; Americans: *r*(55) = -.05, *p* = .74). Directly comparing the values using a Fisher r-to-z transformation indicated that the correlations did not significantly differ between the two groups, z = 1.48, *p* (two-tailed) = .14. No other significant interactions were observed for trial 1. For LDFR, although there was only a trend towards an interaction (p = .07) between right rACC volume and culture, the main effect for right rACC volume reached significance when culture is included in the model; see Table 4.

**Table 3:** Tests of the effects of culture and ROI on CVLT Trial 1. Values are displayed for the contribution of the gray matter volume of the region, the effect on culture, and the interaction of the region x culture (H2).

Region:	*β*	*t*	*p*	Region:	*β*	*t*	*p*
Left Hemisphere				Right Hemisphere			
Lateral Orbitofrontal	0.11	0.76	0.44	Lateral Orbitofrontal	0.07	0.61	0.54
Culture	0.13	0.1	0.91	Culture	-1.28	-1.61	0.24
Lateral Orbitofrontal X Culture	-0.36	-0.28	0.77	Lateral Orbitofrontal X Culture	1.04	0.93	0.35
Rostral Middle	0.06	0.52	0.59	Rostral Middle	-0.01	-0.1	0.92
Culture	0.71	0.82	0.41	Culture	0.27	0.34	0.73
Rostral Middle X Culture	-0.98	-1.09	0.27	Rostral Middle X Culture	-0.51	-0.61	0.54
Superior Frontal	-0.04	-0.32	0.74	Superior Frontal	-0.18	-1.31	0.19
Culture	-2.19	-1.66	0.09	**Culture**	**-3.34**	**-2.26**	**0.02** *
Superior Frontal X Culture	1.95	1.5	0.13	**Superior Frontal X Culture**	**3.06**	**2.07**	**0.04** *
Medial Orbitofrontal	-10	-0.73	0.46	Medial Orbitofrontal	-0.09	-0.72	0.47
Culture	-0.97	-0.87	0.38	Culture	-0.02	-0.02	0.98
Medial Orbitofrontal X Culture	0.76	0.67	0.5	Medial Orbitofrontal X Culture	-0.21	-0.21	0.83
rACC	-0.15	-1.12	0.26	rACC	-0.17	-1.29	0.19
Culture	-0.03	-0.04	0.96	Culture	-0.1	-0.17	0.86
rACC X Culture	-0.17	-0.2	0.83	rACC X Culture	-0.09	-0.14	0.88
cACC	-0.08	-0.57	0.57	cACC	0.13	0.99	0.32
Culture	-0.02	-0.03	0.97	Culture	0.17	0.37	0.7
cACC X Culture	-0.21	-0.27	0.78	cACC X Culture	-0.43	-0.88	0.38

* Indicates significance at p < .05

**Table 4:** Tests of the effects of culture and ROI on CVLT LDFR. Values are displayed for the contribution of the gray matter volume of the region, the effect of culture, and the interaction of the region x culture.

Region Left	*β*	*t*	*p*	Region Right	*β*	*t*	*p*
Lateral Orbitofrontal	-0.06	0.44	0.65	Lateral Orbitofrontal	0.01	0.14	0.88
Culture	-1	-0.75	0.44	Culture	-1.26	-1.1	0.27
Lateral Orbitofrontal X Culture	0.97	0.73	0.46	Lateral Orbitofrontal X Culture	1.24	1.06	0.28
Rostral Middle	-0.07	-0.59	0.55	Rostral Middle	-0.11	-0.9	0.36
Culture	-0.61	-0.67	0.50	Culture	-0.72	-0.86	0.39
Rostral Middle X Culture	0.6	0.64	0.51	Rostral Middle X Culture	0.72	0.84	0.40
Superior Frontal	-0.01	-0.1	0.91	Superior Frontal	0.06	0.43	0.66
Culture	-2.3	-1.7	0.09	Culture	-0.8	-0.51	0.61
Superior Frontal X Culture	2.28	-1.7	0.09	Superior Frontal X Culture	0.79	0.51	0.60
Medial Oribitofrontal	0.02	0.2	0.83	Medial Orbitofrontal	-0.09	-0.72	0.47
Culture	-0.66	-0.57	0.56	Culture	-1.73	-1.7	0.09
Medial Orbitofrontal X Culture	0.62	0.53	0.59	Medial Orbitofrontal X Culture	1.71	1.68	0.09
rACC	0.03	0.25	0.80	**rACC**	**-0.32**	**-2.28**	**0.02** *
Culture	0.5	-0.58	0.56	Culture	-1.08	-1.75	0.08
rACC X Culture	0.47	0.52	0.60	rACC X Culture	1.15	1.77	0.07
cACC	-0.1	-0.71	0.47	cACC	-0.03	-0.26	0.78
Culture	-0.35	-0.43	0.66	Culture	-0.57	-1.19	0.23
cACC X Culture	0.32	3.8	0.70	cACC X Culture	0.58	1.64	0.24

* indicates significance at p < .05

### Exploratory comparisons of brain volumes across cultures

To gain additional understanding of the impact of cultural differences on CVLT performance, we conducted additional exploratory analyses. We first conducted whole-brain analyses on the remaining regions from the DKT atlas, testing interactions between culture and gray matter volume in the regions that were not selected a priori, going beyond the pre-registration. To test this, we used the same approach that was used in the ROI analysis for hypotheses 1 and 2, implementing the same corrections for intracranial volume, running individual models for each brain structure in each hemisphere, and testing for effects for CVLT Trial 1 and LDFR. The regions that reached significance are listed in S2 and S3 Tables. To maintain consistency with the initial ROI analysis, corrections for multiple comparisons were not made.

**Fig 3:**
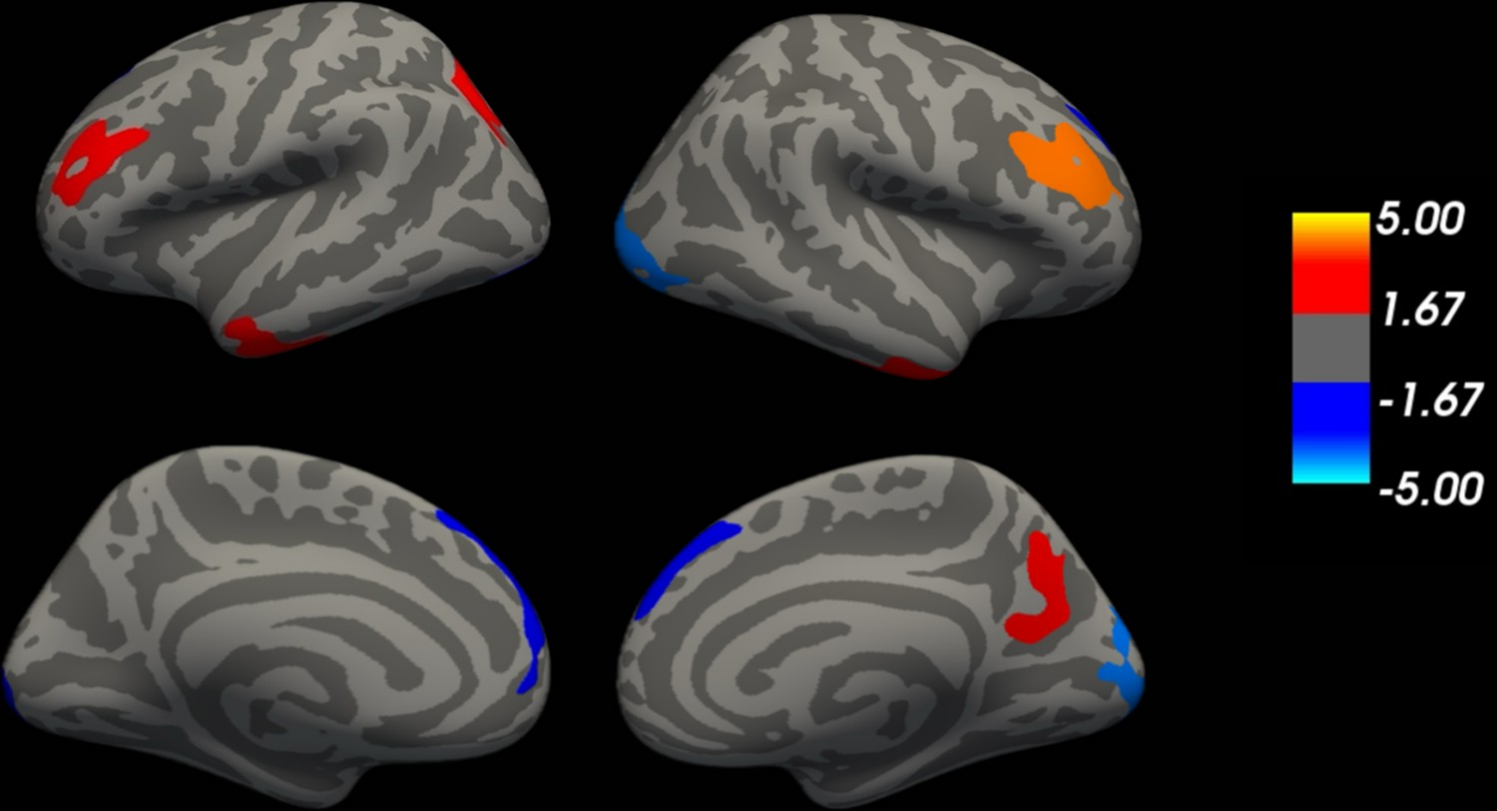
Clusters of differences in gray matter volume among American and Taiwanese young adults. Note: Clusters that are larger for the Taiwanese are displayed in blue and those larger for Americans are displayed in red. For the color coding, a -log10(pvalue) of 5.00 corresponds to p < .0001 and a -log10(pvalue) of 1.67 corresponds to p < .05.

In addition, exploratory tests of cultural differences in volumes using a vertex approach identified some cortical volumes that differed across cultures, as shown in Table 5 and Fig 3. Volumes were larger for Americans compared to Taiwanese in the bilateral superior frontal gyrus and bilateral lateral occipitofrontal gyrus. Taiwanese participants had larger volumes in the bilateral rostral middle frontal gyrus, bilateral inferior temporal gyrus and the right precuneus.

**Table 5:** Whole brain vertex based exploratory analysis (Volume).

	Annotation	Max	VtxMax	Size(mm^2^)	X	Y	Z	CWP
AM>TW	L Superior Frontal	-4.348	121575	1029.39	-7.7	56.3	14.7	0.01
	R Superior Frontal	-4.761	30286	955.29	6.9	35.8	43.1	0.02
	L Lateral Occipital	-5.782	14447	998.72	-16.5	-95.6	-0.9	0.01
	R Lateral Occipital	-4.69	161707	3107.97	20.6	-96.3	-0.4	<0.01
TW>AM	L Rostral Middle Frontal	5.547	56319	1173.43	-32.1	36	20.7	<0.01
	R Rostral Middle Frontal	3.111	115270	1666.36	40	42.7	18.2	<0.01
	L Inferior Temporal	5.573	69886	1045.5	-46.4	-10.2	-28.2	<0.01
	R Inferior Temporal	4.535	92024	1002.63	47.1	-8.7	-27.8	0.01
	R Precuneus	3.755	78098	839.39	8	-64	33.6	0.04

All cluster-wise p-values (CWP) significant at p < .05

## Discussion

This study aimed to address two questions. Hypothesis one posited that higher scores on the CVLT (i.e., learning trial 1; LDFR) would be related to larger volumes in regions of the prefrontal cortex (i.e., superior frontal and rostral middle gyri, lateral orbitofrontal and medial orbitofrontal gyri, rostral and caudal anterior cingulate). The second hypothesis investigated whether there were cultural differences in the relationship between CVLT scores and brain volumes for the prefrontal regions listed above. We focused on twelve anatomically defined prefrontal regions, as prefrontal cortex has been implicated a wide variety of individual differences in terms of memory strategies, including on the CVLT [40], and cultural differences [22, 23, 25].

For hypothesis 1, assessing the relationship between prefrontal volumes and memory, the volume of one region—the rACC–was significantly related to memory performance. These findings build upon those of previous studies that found that thickness in this region bilaterally (labeled rmPFC in their study) predicted higher scores on the LDFR in super-agers [38]. In fact, this region was among several for which cortical thickness was equivalent when comparing super-agers to young adults. This could indicate that memory performance relies on the structural integrity of the region [38]. Interestingly, in contrast to the previous study, we found a *negative* relationship among volumes in the right and left rACC for trial 1 scores; in our study, increased volume was associated with *lower* scores. One critical difference across the studies is that Sun et al [38] studied older adults whereas our sample consists of younger adults. Regions of prefrontal cortex continue to develop into the 20s [40, 80]; the present results may suggest that the thinning of prefrontal cortex in early adulthood is associated with higher levels of memory performance, perhaps through greater use of strategies as the cortex develops. In contrast, loss of volume in prefrontal regions in late adulthood is associated with declines in cognition. Additional research is needed with longitudinal and lifespan samples to investigate the nature of relationships developmentally as well as the consistency of relationships over time within individuals. One difference across the two studies is that Sun et al [38] examined relationships with CVLT scores using a measure of cortical thickness whereas our analyses used volumetric measurements that combine both cortical thickness and surface area [81]. Although one might expect the two measures to have similar relationships with task performance, surface area may dominate volume measures as both are highly correlated with ICV while cortical thickness is not [82], and studies have shown that these two measures have an inverse relationship, particularly in the medial prefrontal cortices where less surface area indicates more thickness and vice versa [83]. Surface expansion is known to be driven by cellular events (e.g., synaptogenesis) during development [84], but the cause of reduction in adulthood is unknown [83], although increases in surface area as a benefit to cognition may be region specific [85]. This could, in part, explain our results for an increase in performance associated with reduced volume. For instance, a negative association between the right rACC and working memory has been demonstrated previously, whereas larger surface area in the left rACC has been related to better neurocognition [86]. Therefore, the different measures could understandably produce varying results and could be domain- and region-specific. Further, thinned cortices in certain cases have been associated with increased cognitive function (for a review, see [87]). Nonetheless, the results contribute to the literature that the volume of the rACC may have implications for memory performance.

For hypothesis 2, that cultures differed in the associations between volumes and memory performance, the relationship between right superior frontal gyrus and CVLT trial 1 performance differed across cultures. Although this interaction emerged as significant in the overall analyses, follow-up analyses to characterize the nature of the interaction did not reach conventional levels of statistical significance. For Taiwanese, there is a trend for a positive relationship between the gray matter volume of right superior frontal cortex and memory performance. This pattern contrasts with that of the Americans, for which there was not a clear trend for a relationship between gray matter volume of right superior frontal cortex and memory performance. The different patterns across the two groups should be interpreted with caution due to the lack of significant differences in follow-up analyses of the correlation values. If the pattern is replicated in larger samples or samples with more variation in prefrontal gray matter volumes (e.g., older adults), the finding could indicate that larger gray matter volumes in right superior frontal gyrus are associated with better performance in Taiwanese but not in Americans. Potential cultural differences in the relationship between gray matter and trial 1 scores could reflect variation in cognitive strategies. For instance, given that trial 1 of the CVLT is a learning task with a short delay before retrieval, strongly associated with attention [88], this finding could potentially reflect the differences these two cultures demonstrate in allocating attention. For instance, in this study we considered the superior frontal areas a part of the dlPFC, which has been associated with organization of information in working memory, and subsequent memory performance, in particular, under retrieval conditions that target memory for association between items (for review; see [89]). Because Americans perform better on average than Taiwanese on the 1^st^ learning trial, it may be the case that brain regions linked to attention and organization play a more substantial role for the Taiwanese, such that those with larger volumes in these regions perform better than those with smaller volumes, whereas the reverse is true for Americans. Furthermore, because this region is a part of the dlPFC, the region contributes to a variety of memory strategies and performance on the CVLT [38, 40]. Future research could further probe the strategic aspects of memory using the CVLT, explicitly assessing the use of categories as a recall strategy. Such analyses may be most promising in comparisons of older adults, based on prior findings cultural difference in the use of a clustering strategy in free recall memory emerged more strongly in comparisons of older, more than younger, American and Chinese adults [13]. Research with older adults may also better support detection of relationships between volumes and performance on the LDFR portion of the CVLT, as scores were high for the present samples of young adults.

Although the present study focused on relationships between gray matter volumes and performance on a neuropsychological task investigating memory, past cross-cultural studies largely focused on comparing the volume of regions without considering the relationship to task performance. Our exploratory analyses comparing volumes across cultures–apart from considering relationships with CVLT scores–found that the volume of bilateral superior frontal gyrus was larger for Americans than Taiwanese converges with prior comparisons of Westerners and East Asians [23, 25]. Similarly, our finding that the volume of right rACC was larger in Taiwanese than Americans converges with prior findings regarding the cingulate [23]. Cultural differences in bilateral rostral middle frontal cortex, however, did not emerge in these prior papers. Additional exploratory analysis using a vertex analysis approach replicated the above findings, in that American young adults have significantly more gray matter volume in bilateral superior frontal cortex and Taiwanese have larger volumes in bilateral rostral middle frontal cortex. Outside of frontal regions, Americans had larger volumes in bilateral lateral occipital gyrus compared to East Asians, in line with prior findings [90]. Converging with prior studies showing that East Asians have larger volumes in temporal areas than Americans [23, 25], East Asians had larger volumes in bilateral inferior temporal cortex, as well as in right precuneus (See Table 5). This overall pattern of convergence in patterns across studies occurs despite methodological differences (e.g., volumetric analyses vs. VBM). Future comparisons of samples drawn from multiple Eastern and Western populations and use of multiple methods would be helpful to assess the robustness and consistency of volumetric differences across specific regions, particularly in terms of the dissociation in the effects of culture on frontal versus temporal and parietal regions. In addition, studies comparing cultures across age groups (e.g., [22]) would allow for comparisons across a wide range of volumes, as these can be differentially impacted by aging.

Methodologically, the Freesurfer parcellations offer some advantages in that the method is immune to differences in brain shape across different cultural groups. There are, however, several limitations to the study. Although the sample sizes are on par with prior studies comparing structural volumes across cultures (n = 50–60 participants per group), they could still be considered small for the comparison of effects related to performance and culture (e.g., [91]). Furthermore, we did not adjust p values for multiple comparisons, potentially running the risk of false positives. Replication of results in larger samples, as well as samples with more variability in gray matter volume and performance on the CVLT, such as older adults, will be important to validate the results. Although samples were well-matched on many dimensions, including the lack of psychiatric diagnoses, medications for such diagnoses, and alcohol/drug problems, there may be cultural differences not picked up by these questions. For example, Americans tend to report higher levels of depression, anxiety, and alcohol and drug use than East Asian samples, even if these did not rise to the level of exclusions for our study. In addition, most of the Taiwanese sample would be expected to be bilingual, whereas only seven of the American participants reported high levels of proficiency in a 2^nd^ language. Although we did not assess these factors, body mass index (BMI) would be expected to be higher in Americans than Taiwanese, and more of the males in the Taiwan sample would be expected to have military experience (although note that the required service can occur at varied ages throughout ones 20s and may involve service in the public sector rather than military training).

## Conclusions

The findings provide evidence that the volume of the rACC may be associated with memory performance. Specifically, volume in the bilateral rACC predicted lower performance for the CVLT trial 1. Culture moderates the relationship between the volume of the right superior frontal gyrus and performance on trial 1, with a trend for a positive relationship for Taiwanese but not Americans (although further research is needed to characterize the nature of the cultural differences). Nonetheless, these findings are consistent with previous literature implicating culture in different relationships between behavior and brain structure [29, 30]. We extend prior work focused on individual differences in social identity and orientation to performance on neuropsychological tasks of cognitive function.

## Supporting information

S1 Table Exploratory comparison of cultural groups’ performance on other measures from the CVLT. (DOCX)

S2 Table Significant interactions from a whole brain exploratory analysis of the interaction between culture and volume on CVLT Trial 1 (H1). (DOCX)

S3 Table Significant effects from a whole brain exploratory analysis of the interaction between culture and volume on CVLT LDFR (H2). (DOCX)
