# Supplementary material for: Culture, prefrontal volume, and memory
Source: PLoS One. 2024 Mar 29;19(3):e0298235. doi: 10.1371/journal.pone.0298235 (PMC10980194; doi:10.1371/journal.pone.0298235)
Supplement: S1 Table — (DOCX) [file pone.0298235.s001.docx]

| **Supplementary Table 1**  **Exploratory comparison of cultural groups’ performance on other measures from the CVLT** | | | | | | | |
| --- | --- | --- | --- | --- | --- | --- | --- |
|  | Culture | | *N* | *M* | *SD* | *t* | *p* |
| CVLT Trial 2 | | AM | 58 | 12.33 | 2.16 | 1.73 | .08 |
|  |  | TW | 57 | 11.58 | 2.48 |  |  |
| CVLT Trial 3 | | AM | 57 | 13.84 | 2.02 | 1.89 | .06 |
|  |  | TW | 57 | 13.11 | 2.15 |  |  |
| CVLT Trial 4 | | AM | 58 | 14.38 | 1.65 | .34 | .73 |
|  |  | TW | 55 | 14.27 | 1.65 |  |  |
| CVLT Trial 5 | | AM | 57 | 14.74 | 1.52 | .18 | .86 |
|  |  | TW | 57 | 14.68 | 1.58 |  |  |
| CVLT List B | | AM | 58 | 8.43 | 2.41 | 3.03 | .003* |
|  |  | TW | 54 | 7.15 | 2.03 |  |  |
| CVLT SDFR | | AM | 58 | 13.67 | 1.98 | .66 | .51 |
|  |  | TW | 56 | 13.38 | 2.79 |  |  |
| CVLT SDCR | | AM | 57 | 14.21 | 1.92 | .51 | .61 |
|  |  | TW | 56 | 14.02 | 2.11 |  |  |
| CVLT LDCR | | AM | 57 | 14.44 | 1.80 | -.22 | .83 |
|  |  | TW | 55 | 14.51 | 1.55 |  |  |
| CVLT Recognition | | AM | 57 | 15.60 | .75 | -.15 | .88 |
|  |  | TW | 55 | 15.62 | .73 |  |  |

Notes: SD = Short-delay; LD = Long-delay; FR = Free recall; CR = Cued Recall

AM = American; TW = Taiwanese;

* = significant at p<.05
