# Supplementary material for: Culture, prefrontal volume, and memory
Source: PLoS One. 2024 Mar 29;19(3):e0298235. doi: 10.1371/journal.pone.0298235 (PMC10980194; doi:10.1371/journal.pone.0298235)
Supplement: S2 Table — (DOCX) [file pone.0298235.s002.docx]

**Supplementary Table 2:** Significant interactions from a whole brain exploratory analysis of the

interaction between culture and volume on CVLT Trial 1 (H1)

| Volume |  |  | *β* | *t* | | *p* | |  |
| --- | --- | --- | --- | --- | --- | --- | --- | --- |
|  |  |  |  |  | |  | |  |
| R entorhinal | |  | -.39 | -3.05 | | <.01 | |  |
| Culture |  |  | -1.17 | -2.52 | | 0.01 | |  |
| R Entorhinal X Culture | |  | .96 | 2.02 | | 0.04 | |  |
|  |  |  |  |  | |  | |  |
| L Inferior Parietal | |  | .24 | 2.03 | | 0.04 | |  |
| Culture |  |  | 1.35 | 1.84 | | 0.07 | |  |
| L Inferior Parietal X Culture | | | -1.67 | -2.22 | | 0.03 | |  |
|  |  |  |  |  | |  | |  |
| R Inferior Parietal | |  | .40 | 2.87 | | <.01 | |  |
| Culture |  |  | 2.35 | 2.76 | | <.01 | |  |
| R Inferior Parietal X Culture | | | -2.66 | -3.08 | | <.01 | |  |
|  |  |  |  |  | |  | |  |
| L Middle Temporal | |  | .33 | 2.57 | | 0.01 | |  |
| Culture |  |  | 2.10 | 2.2 | | 0.03 | |  |
| L Middle Temporal X Culture | | | -2.43 | -2.49 | | 0.01 | |  |
|  |  |  |  |  | |  | |  |
| R Middle Temporal | |  | .35 | 2.9 | | <.01 | |  |
| Culture |  |  | 2.57 | 2.41 | | 0.02 | |  |
| R Middle temporal X Culture | | | -2.88 | -2.68 | | 0.01 | |  |
|  |  |  |  |  | |  | |  |
| R Pars Triangularis | |  | .23 | 1.87 | | 0.06 | |  |
| Culture |  |  | 1.19 | 2.26 | | 0.03 | |  |
| R Pars Triangularis X Culture | | | -1.49 | -2.79 | | 0.01 | |  |
|  |  | |  |  |  | |  | |
