# Supplementary material for: Culture, prefrontal volume, and memory
Source: PLoS One. 2024 Mar 29;19(3):e0298235. doi: 10.1371/journal.pone.0298235 (PMC10980194; doi:10.1371/journal.pone.0298235)
Supplement: S3 Table — (DOCX) [file pone.0298235.s003.docx]

**Supplementary Table 3**

*Significant effects from a whole brain exploratory analysis of the interaction between*

*culture and volume on CVLT LDFR (H2)*

| Volume |  | *β* | *t* | *p* |
| --- | --- | --- | --- | --- |
|  |  |  |  |  |
| R Pars Triangularis | | .17 | 1.31 | 0.19 |
| Culture |  | 1.34 | 2.45 | 0.02 |
| R Pars Triangularis X Culture | | -1.42 | -2.55 | 0.01 |
